# PReS13-SPK-1082: Catastrophic antiphospholipid syndrome

**DOI:** 10.1186/1546-0096-11-S2-I10

**Published:** 2013-12-05

**Authors:** R Cervera

**Affiliations:** 1 Autoimmune Diseases, Hospital Clínic of Barcelona, Barcelona, Spain

## 

In the 1980's, isolated case reports appeared in the world literature documenting patients who appeared to suffer from an often fatal complication associated with the demonstration of antiphospholipid antibodies (aPL). The clinical picture comprised widespread multi-organ thrombosis and consequent organ failure and was referred to by the authors as a "devastating non-inflammatory vasculopathy","occlusive vasculopathy" or "acute disseminated coagulopathy-vasculopathy" when describing individual cases. In 1992, ten patients with this unusual condition were first reviewed and, in an attempt to define its acuteness and severity, the eponym "catastrophic" was attached to this variant of the antiphospholipid syndrome (APS).

Although less than 1% of patients with the APS develop this complication, its potentially lethal outcome, despite all recommended therapies, emphasizes its importance in clinical medicine today. The majority of these patients end up in Intensive Care Units (ICU) with multi-organ failure and, unless the condition is considered in the differential diagnosis by the attending physicians, it may be completely missed with a disastrous outcome for the patients.

The rarity of the syndrome makes it extraordinarily difficult to study in any systematic way. In order to put together all the published case reports as well as the new diagnosed cases from all over the world, an International Registry of patients with catastrophic APS was created in 2000 by the *European Forum on Antiphospholipid Antibodies *("CAPS Registry"). The initial results of the project have been already presented in several original papers. Currently, it documents the entire clinical, laboratory and therapeutic data of more than 400 patients whose data has been fully registered and it is expected that the periodical analysis of these data will allow us to increase our knowledge of this condition. The purpose of this presentation is to focus on the current management of these patients and some of the potential new therapeutic approaches.

## Disclosure of interest

None declared.

